# People with psychogenic non-epileptic seizures: A South African perspective

**DOI:** 10.4102/ajod.v4i1.176

**Published:** 2015-07-10

**Authors:** Chrisma Pretorius, Gretha Cronje

**Affiliations:** 1 Department of Psychology, Stellenbosch University, South Africa

## Abstract

**Background:**

Psychogenic non-epileptic seizures (PNES) is a disabling disorder which has a negative effect on the quality of life of individuals with PNES. A clear understanding of the disorder is necessary, however, to date, research about PNES in South Africa is limited.

**Objectives:**

The aims of this study were to explore the demographic variables of individuals with PNES in South Africa, to review the available body of research on PNES, and to compare it with our results.

**Method:**

Twenty-two people with PNES, with confirmed video EEG, were recruited by means of convenience sampling from two hospitals. Descriptive statistics were used to describe the demographic variables of the participants.

**Results:**

Internationally comparable results revealed misdiagnoses and low treatment delivery amongst a primarily female population.

**Conclusion:**

This study provided greater insight into individuals with PNES in South Africa, highlighting the need for more information, support, effective treatment and accurate diagnosis of PNES.

## Introduction

Psychogenic non-epileptic seizures (PNES) is described as paroxysmal behaviour patterns that mimic epileptic seizures (ES) but are not associated with abnormal electrical discharges in the brain (Bodde *et al.* 2009). These episodes are seen as ‘a response to psychological or social distress, which occurs when alternative coping mechanisms are inadequate or have been exhausted’ (Plug, Sharrack & Reuber 2009:994). It is also believed that PNES arises from an unconscious process and that these individuals do not deliberately fake the seizures (Goldstein & Mellers 2006). For this reason, it cannot be regarded as malingering (Gross 1983).

PNES is more commonly (e.g., 80%) observed in women (Bora *et al.* 2011; Deveci *et al.* 2007; Reuber *et al.* 2003; Szaflarski *et al.* 2000; Uliaszek, Prensky & Baslet 2012). The occurrence of PNES appears to peak during the second and third decade of life (Deveci *et al.* 2007; Strutt *et al.* 2011). Studies focussing on children and adolescents with PNES indicated a mean age of 14 years (Hempel, Doss & Adams 2010). Rare incidences of PNES have been identified in individuals younger than 4 years (Reuber 2008) and older than 65 years (Szaflarski *et al.* 2000). People with PNES typically have an older age of onset than people with epilepsy (ES) (Cragar *et al.* 2005).

The prevalence rate of PNES in the US and Western Europe is estimated to be between two to 33 per 100 000 (Benbadis & Hauser 2000). Data from epilepsy centres report a much higher prevalence rate of PNES (Woollacott *et al.* 2009), because of the general misdiagnosis of PNES as ES and the resistance of people with PNES to anti-epileptic drugs (AEDs) (Bodde *et al.* 2009). Research indicates that 20% to 30% of people referred to epilepsy centres for examination of seizure disorders have PNES (Benbadis & Hauser 2000; Griffith & Szaflarski 2010; Martin *et al.* 1998). The incidence and prevalence rates of PNES in South Africa are still unknown because no epidemiological studies on this condition have been conducted in South Africa. However, according to a neurologist at the Epilepsy Unit of the Constantiaberg Medi-Clinic in South Africa, several individuals per month are diagnosed with PNES at their facilities (J. Butler, pers. comm., 09 March 2011).

Despite the increase in published knowledge about PNES, the average period between the manifestation of the first seizure and a correct diagnosis remains unacceptably long at approximately seven years (Cragar *et al.* 2002; Jones *et al.* 2010; LaFrance Jr. 2008). Diagnosing PNES is difficult (Reuber & Elger 2003). Abnormal electroencephalogram (EEG) patterns during the inter-ictal phase of seizures have been reported to be unhelpful in distinguishing PNES from ES (Benbadis & LaFrance Jr. 2010). According to Brown *et al.* (2011), the main reason for this is:

1. over-interpretation of non-specific EEG changes (it is worth noting that this is specific to practice in the USA)
2. the fact that a normal inter-ictal EEG does not exclude epilepsy, limiting the diagnostic usefulness of outpatient EEG.

However, the introduction of new diagnostic techniques such as the simultaneous video EEG (VEEG) in the late 20th century enabled physicians to rule out cardiac or neurological causes of seizure-like events (Hamilton *et al.* 2010). Therefore, proper knowledge of PNES symptomatology is important for early screening of these individuals for VEEG recording and the correct interpretation following the examination (Mostacci *et al.* 2011). Although no single clinical feature or observation is pathogenic of PNES (Hoerth *et al.* 2008; Reuber & Elge 2003) certain behaviours are associated strongly with PNES (Benbadis & LaFrance Jr. 2010). The following features are clinically useful to raise the question of whether the nature of a seizure may not be epileptic, but rather psychogenic:

- longer seizure duration (Cragar *et al.* 2002; Reuber & Elger 2003)
- resistance to AEDs
- high frequency of seizures (e.g., daily) (Benbadis & LaFrance Jr. 2010)
- ictal stuttering (Benbadis & LaFrance Jr. 2010; Hoerth *et al.* 2008)
- pelvic thrusting (Benbadis & LaFrance Jr. 2010; Cragar *et al.* 2002; Hoerth *et al.* 2008; Mostacci *et al.* 2011)
- preserved consciousness (Benbadis & LaFrance Jr. 2010; Mostacci *et al.* 2011).

Complicating the diagnosis is the fact that in 10% to 20% of people with PNES, ES and PNES coexist (Griffith & Szaflarski 2010; Lezak, Howieson & Loring 2004), and also the fact that VEEG monitoring is expensive and not always available (Hoerth *et al.* 2008; Reuber & Elger 2003). However, PNES, when misdiagnosed, is costly to people with PNES, the health care system and society. It may lead to:

- prolonged treatment with AEDs for what is mistakenly thought to be epilepsy (Jones *et al.* 2010; Reuber *et al.* 2003)
- a delay of appropriate psychological treatment (Bodde *et al.* 2009; Hoerth *et al.* 2008)
- unnecessary hospitalisation
- frequent use of health care resources (Asmussen *et al.* 2009)
- unemployment (Hamilton *et al.* 2010)
- a negative effect on social development (Bodde *et al.* 2007).

People with PNES, as a group, are very heterogeneous and PNES does not have a single psychological aetiology (Bodde *et al.* 2009; Lezak *et al.* 2004; Reuber *et al.* 2007).

Differences with regard to psychosocial, psychological, and organic factors have been identified (Baslet, Roiko & Prensky 2010; Lezak *et al.* 2004). The most common predisposing factors of PNES reported are:

- trauma
- family dysfunction
- psychiatric comorbidity
- coping mechanisms.

Traumatic events most commonly reported are:

- childhood sexual or physical abuse (Bowman 2010)
- stressful life events
- severe physical illness (Turner *et al.* 2011)
- bereavement (Bora *et al.* 2011).

People with PNES viewed their families as being more dysfunctional than did people with ES, particularly with regard to communication, affective involvement, and conflict (Bowman 2010; LaFrance Jr. 2008; Reuber *et al.* 2007). Most of the people with PNES have comorbid psychiatric disorders of which depression and anxiety are the most commonly reported (Asmussen *et al.* 2009; Goldstein & Mellers 2006; Mercer, Martin & Reuber 2010). People with PNES also generally make use of avoidance coping strategies to deal with everyday problems (Cronje & Pretorius 2013; Reuber 2008).

The literature suggests that sociocultural factors that are strongly associated with the development of PNES in Western countries include sexual or physical abuse (Bowman 2010), trauma (Hingray *et al.* 2011), and conflict in the family (LaFrance Jr. 2008; Reuber *et al.* 2007). In Eastern countries, however, research reveals that gender-specific inequalities (Bora *et al.* 2011; Deveci *et al.* 2007), education (Bora *et al.* 2011; Hingray *et al.* 2011), socioeconomic status (Deveci *et al.* 2007), and dysfunctional family structures (Dhanaraj *et al.* 2005) are the sociocultural factors that play the greatest role in the development of PNES. However, research that focuses more specifically on these cultural differences is still needed. South Africa is a multicultural country with numerous ethnic groups. Several of the abovementioned sociocultural factors might, therefore, play a role in the development of PNES in South African individuals.

Although different predisposing factors for the development of PNES have been reported, researchers are in agreement that PNES does have a negative effect on the health-related quality of life (HRQOL) of individuals (Cronje & Pretorius 2013; Mercer *et al.* 2010). Previous research indicates that HRQOL is significantly lower in people with PNES than it is in people with ES (Al Marzooqi *et al.* 2004; Testa *et al.* 2007) and in the general population (Cronje & Pretorius 2013; Mercer *et al.* 2010; Uliaszek *et al.* 2012). Strutt *et al.* (2011) reported that the individuals, with PNES, in their study believed that their low physical, emotional and social functioning was a direct result of their PNES condition. These findings highlight the psychological and physical problems (e.g., difficulties with daily activities or work) that people with PNES experience.

According to Brown *et al.* (2011), one of the benchmarks for epilepsy research of the National Institute of Neurological Disorders and Stroke (NINDS) is to develop treatments for PNES, because of the incidence and prevalence of the disorder and the lack of treatment efficacy data. The most common treatment plans, based on the PNES aetiology, include:

- cognitive-behavioural therapy (CBT)
- psychodynamic orientated psychotherapy
- group psychotherapy
- family therapy
- a multidisciplinary approach (LaFrance Jr. & Bjørnaes 2010; LaFrance Jr. *et al.* 2014).

In the literature, no consensus exists about the types of treatment that may be most effective for treating PNES (Zaroff *et al.* 2004). It also does not seem possible to develop a ‘one-size fits all’ treatment, because of the multifactorial aetiology of this group (Reuber 2008). However, in a pilot randomised controlled trial study in the United Kingdom, it has been found the frequency of seizures in people with PNES were reduced more effectively with treatment with cognitive-behavioural therapy than standard medical care on its own. (Goldstein *et al.* 2010). These findings are forming the basis for a major multicentre trial now underway in the United Kingdom. Furthermore, a recent study of La France *et al*. (2014) indicated that PNES can be effectively treated with manualised CBT. Although people with PNES are some of the most challenging to treat in medical practice (Jones *et al.* 2010), research demonstrates that PNES is a treatable disorder (LaFrance Jr. & Bjørnaes 2010; LaFrance Jr. *et al.* 2014). Although limited research has been undertaken on long-term prognosis, studies consistently report that a third to a fourth of people with PNES become chronic PNES sufferers (Bodde *et al.* 2009). Possible reasons for this may be the differences in psychopathology; for some people with PNES intensive and prolonged therapy is necessary for a favourable prognosis (Bodde *et al.* 2009; Cragar *et al.* 2005). However, it should be noted that information about more global measures of outcomes in people with PNES is lacking (Martin *et al.* 1998).

PNES is as disabling as epilepsy (Al Marzooqi *et al.* 2004; LaFrance Jr. *et al.* 2014; Testa *et al.* 2007) and has a negative effect on the HRQOL of individuals in South Africa (Cronje & Pretorius 2013).

Although the prevalence rate of PNES is unknown in South Africa, PNES is commonly diagnosed at epilepsy-monitoring units, according to a neurologist at the Epilepsy Unit of the Constantiaberg Medi-Clinic in South Africa (J. Butler, pers. comm., 09 March 2011). New investigatory techniques make it possible to distinguish between epileptic and non-epileptic seizures. However, access to appropriate diagnostic facilities and health care workers capable of making the diagnosis is limited, resulting in misdiagnosis that is costly to the patient and also to the health care system. From the literature reviewed, it is clear that an understanding of the disorder is necessary for people with PNES to accept the diagnosis and for health care workers to treat these patients successfully. However, to date research about PNES in South Africa is limited. Therefore, the primary aim of this study was to explore the demographic variables of people with PNES in order to gain a better understanding of people with PNES in the South African context.

## Research method and design

### Participants, procedure, data analysis and ethical considerations

By means of convenience sampling, 22 participants were recruited to participate in this study. The participants had to be South African citizens and 14 years of age or older. The eligibility criterion for each PNES participant was a confirmed diagnosis of PNES by an experienced neurologist, based on the results of VEEG. People with PNES and comorbid epilepsy were excluded from the study.

Data collection took place over a period of nine months. People with PNES attending the Epilepsy Unit at the Constantiaberg Medi-Clinic, or the Department of Neurology at the Tygerberg Hospital, for assessment or treatment were informed by the neurologist of the opportunity to participate in the study.

These hospitals were selected because access to participants in both these regions is convenient, and both hospitals have specialised VEEG equipment to diagnose people with PNES.

A demographic questionnaire was used to obtain the information required for descriptive purposes and to determine demographical variables of the participants. The demographic questionnaire was developed by reviewing demographic information from previous studies and compiling a questionnaire with the typical questions. Descriptive statistics were used to describe the basic features of the data in this study, and provide simple summaries about the sample and about the observations that have been made. Participants had to give written informed consent to participate in this study. This research was explorative and formed part of a larger study. Ethical approval for this study was obtained from the Health Research Ethics Committee at Stellenbosch University (Protocol number: N11/08/267).

## Results

In total, 25 individuals with a confirmed VEEG diagnosis of PNES were referred to the researcher during the data collection period. Most of the people with PNES (*n* = 21) were referred by the neurologist at the Epilepsy Unit of the Constantiaberg Medi-Clinic. Of the 25 people with PNES that were referred to the researcher, only three people chose not to participate in the study, which means that the study had a participation rate of 88%.

Most of the PNES participants (77%) were female, whereas only 23% (5 of the 22) were male. The mean age of the PNES group was 32.77 years (with a SD of 14.40). The age distribution of this sample was bimodal, where 32% of the sample was between the ages of 10 and 20 years and 27% of the sample was between 40 and 50 years. One of the reasons for this distribution may be that the sample consisted of a combination of adolescents and adults (people of 14 years in age and older).

Half of the participants’ home language was Afrikaans, and the remainder were English speaking. Most of the people with PNES in this study (68%) were Caucasian, and the remainder of the sample was mixed-race (32%). The ethnicity categories were included in this study only as a control measure to determine how closely the distribution of the participants reflects the diverse population of South Africa in general. However, the sample size of this study is too small to draw any conclusion about the population distribution of people with PNES in South Africa. It is important to note that the social and ethnic mix of the study sample is certainly not representative of the general South African population. A possible reason for this may be because the majority of the sample (84%) was recruited from private health care services.

The rest of the demographic information of the PNES group is summarised in Table 1.

**TABLE 1:** Demographic information of people with psychogenic non-epileptic seizures (*N* = 22).

Variables	*n*	%
**Marital status**		
Single	10	45
Married	11	50
Divorced	1	5
**Education level**		
Before Grade 12	11	50
Grade 12	5	23
Tertiary diploma	2	9
Degree at university	3	14
**Employment status**		
Unemployed	6	27
Full-time employed	8	36
Part-time employed	-	-
Homemaker	1	5
Medically retired	1	5
Retired	-	-
Student	6	27
**Household income**		
Low income	4	18
Middle-income	13	59
High income	5	23
**Have you been diagnosed with a psychiatric disorder in the past year?**		
Yes	6	27
No	16	73
**Have you been diagnosed with epilepsy before?**		
Yes	7	32
No	15	68
**How long did it take before you were diagnosed with PNES?**		
Less than 1 year	12	54
1-2 years	3	14
2-3 years	2	9
3-4 years	2	9
4-7 years	-	-
More than 7 years	3	14
**How often do you have seizures?**		
More than once a day	8	36
Once every day	3	14
More than once a week	6	27
Once a week	2	9
Every 2nd week	2	9
Once a month	-	-
Once a year	-	-
**Are you currently receiving any treatment?**		
Yes	8	36
No	14	64
**What type of treatment are you receiving?**		
Psychodynamic therapy	6	27
Cognitive-behavioural therapy	1	5
Anxiety medication	1	5
**Would you like to be part of a support group?**		
Yes	15	68
No	7	32
**Would you like to receive your support individually or in a group?**		
Individually	3	14
Group	11	50
**What type of other support would you like?**		
More information	7	32
Telephonic	3	14
Website	5	23
Talk to someone who had PNES	12	54

PNES, psychogenic non-epileptic seizures.

Table 1 reflects that the majority of the participants were married (50%). Seven of the PNES participants were still at school. Of the PNES participants that were not at school or studying, only 53% were employed full-time, whereas 40% were either unemployed or medically retired. Most of the PNES participants viewed their household income to fall within the middle-income group. Only 32% (7 of 22) of the participants had been diagnosed with epilepsy before. For half of the PNES participants, it took less than a year before they were diagnosed with PNES. For 32% of the PNES group, it took between one and four years. Only 14% of the PNES participants indicated that it had taken more than seven years to be diagnosed with PNES. Half of the PNES group had one or more than one seizure per day. Most of the PNES participants indicated that they were not receiving any psychological treatment. Those who were receiving treatment were mostly receiving psychodynamic therapy. More than half of the PNES participants indicated that they would like to be part of a support group and receive support from someone who has previously been diagnosed with PNES.

## Discussion

The majority of the PNES sample of this study consisted of women. This tendency is in line with other studies that reported that PNES is gender related and more commonly observed in women (Bora *et al.* 2011; Deveci *et al.* 2007). The exact nature of the close relationship between females and PNES is not yet well understood (Schmitz 2010). Oto *et al.* (2005) found no significant gender differences between social and aetiological factors in their study of people with PNES.

Congruent with other reports in the literature, the PNES participants in our sample had low education levels. Most of the PNES participants in our study (excluding the seven participants that were still at school) had an education level of Grade 12 or lower. Previous research indicates that sociocultural factors such as poor education (Bora *et al.* 2011; Deveci *et al.* 2007) may play a role in the development of PNES.

In contrast to Deveci *et al.* (2007), who indicate that low socioeconomic status may play a role in the development of PNES, most of the PNES participants in our study (82%) viewed themselves as being within the middle or high income bracket. One of the reasons for the relatively high socioeconomic status of our PNES group may be that most (84%) of the participants were recruited from a private hospital (the Constantiaberg Medi-Clinic). Therefore, these results should be interpreted with care and not be seen as an indication of the general PNES population in South Africa. Most of the South African population does not have access to private health care or medical aid funds and is, therefore, incapable of affording access to private hospitals with the necessary VEEG equipment. At the time of the study the Tygerberg Hospital had only one VEEG monitoring unit, whereas the Epilepsy Unit at the Constantiaberg Medi-Clinic had several VEEG monitoring units.

Studies indicate that the average period between seizure manifestation and correct diagnosis is about seven years (Cragar *et al.* 2002; Jones *et al.* 2010; LaFrance Jr. 2008). However, in this study, only 14% of the PNES participants indicated that it took seven years or more, after their first seizure manifestation, before they were diagnosed with PNES. Most of the PNES participants (55%) received the correct diagnosis of PNES within one year of their first seizure. For the rest of the participants (31%), it took between one to four years before they received the correct diagnosis. A possible reason for the earlier diagnosis in our study may be that the hospital where most of the participants were recruited (the Constantiaberg Medi-Clinic) has several VEEG monitoring units with sufficient equipment to confirm a correct diagnosis of PNES. It may not be the case at other hospitals or epilepsy units in South Africa as VEEG monitoring is costly and not available countrywide (Hoerth *et al.* 2008; Reuber & Elger 2003). It is also possible that there may be people with PNES in South Africa for whom a longer period (more than seven years) has lapsed before a correct diagnosis was made, as our study made no provision for people with PNES who were not referred to epilepsy units (i.e., they only saw a general practitioner, because of travel difficulties or financial limitations). Research found that, without a VEEG, the possibility of a definite diagnosis of PNES is only 50%, because certain seizure types (e.g., frontal lobe seizures) may mimic PNES symptoms (LaFrance Jr. 2008). Therefore, an epilepsy-monitoring unit is usually necessary to distinguish epilepsy from PNES (Szaflarski *et al.* 2000).

Although our results indicate a shorter period between seizure manifestation and the diagnosis of PNES than previous studies, a third of the PNES participants were initially misdiagnosed with epilepsy. Researchers indicate that the misdiagnosis of PNES (Hamilton *et al.* 2010; Martin *et al.* 1998) increases the medical burden of PNES on society. Almost half of the PNES group (45%) only received the correct diagnosis more than one year after their first seizure. The early diagnosis of PNES is not only important to reduce unnecessary medication costs (such as AEDs). It is also important because a proper diagnosis is the first step in PNES treatment and the outcome is better in people with a shorter history of PNES (Cragar *et al.* 2002; Jones *et al.* 2010).

In line with previous research (Jones *et al.* 2010), only a third of the PNES participants indicated that they were receiving psychological treatment at the time of completing the research survey. Possible barriers to appropriate psychological treatment may result from the following factors:

- a patient's refusal to accept the diagnosis (Bodde *et al.* 2007)
- poor understanding of PNES by health care workers
- over emphasis on the seizures
- limited recognition of the psychological aspects
- transportation limitations
- lack of resources in rural areas (Jones *et al.* 2010; LaFrance Jr. 2008; LaFrance *et al*. 2014).

The PNES participants in our study showed a high frequency of events which are typical of PNES, as reported in previous literature (Benbadis & LaFrance Jr. 2010). Half of the participants had one or more than one seizure per day, whilst 36% had at least one seizure per week. High PNES frequency does have a negative influence on HRQOL (Lawton *et al.* 2008). People with PNES experience bodily pain, and difficulties with daily and work related activities because of the high frequency of seizures. In our study 40% of the people with PNES were unemployed or medically retired when they were diagnosed with PNES.

Research indicates that psycho-education and a clear understanding of the disorder is necessary for people with PNES to accept the diagnosis and to treat these people successfully (Bodde *et al.* 2007; Duncan, Razvi & Mulhern 2011; Zaroff *et al.* 2004). A third of the PNES participants indicated that they would like more information about PNES. Most of the PNES participants (68%) also indicated that they would like to be part of a support group. From these results, it seems that there is a need for people with PNES to feel part of a group. Zaroff *et al.* (2004) reported that placing people with PNES in a group allowed them to feel that PNES is not as uncommon as they might think. To provide these people with appropriate support, it may be beneficial for the neurologist or psychiatrist to provide them with enough information and the contact details of a PNES support group (if available) when the diagnosis is communicated to the person.

### Limitations

Although the sample size seems to correspond with the low prevalence rate of PNES, and sample sizes in previous PNES research that generally vary between 20 and 30 participants (Bodde *et al.* 2009), the results of the study need to be interpreted with caution. The current sample consisted of a convenience sample of people with PNES. Consequently, the findings of the study cannot be generalised validly beyond this particular population.

The fact that the results represent the experience of people with PNES from only two epilepsy centres (both of which are in the Western Cape region of South Africa) may reflect biases of the epilepsy practices involved at these centres, and can be seen as a limitation of the study. Furthermore, it is important to note that the social and ethnic mix of the study sample is not representative of the general South African population. This may be because the majority of the sample was recruited from private health care services. Thus, generalising the results of the current study to the general South African population of people with PNES would be inappropriate, because this study is more reflective of a particular niche within the South African health care system, than representative of the South African health care system in general. However, the researchers would like to note that the people with PNES recruited from the Epilepsy Unit of the Constantiaberg Medi-Clinic were from across the country, as that unit is the best equipped in South Africa to diagnose PNES. It should furthermore be noted that there are very few epilepsy centres available in South Africa.

## Conclusion

In general this study supports the demographic results of previous international studies. It is evident from this study that PNES has a severe, negative effect on all emotional, social and physical aspects of the individual. It also highlights that people with PNES represent a public health problem resulting from the diagnostic difficulty, the poor prognosis and their unemployment status. The average medical cost in the South African context is unknown, but given that these individuals represent a proportion of people seen for seizure disorders by general practitioners, psychiatrists, psychologists, and in particular neurologists, and taking into account the average length of time before a patient is diagnosed with PNES, the burden of PNES on these individuals, medical aids and, also, the health care system of the country, may be substantial and a research avenue worth investigating.

Few research funding initiatives have been directed towards understanding and treating PNES in South Africa. It is expected that this study will contribute to raising more awareness amongst clinicians in South Africa about considering PNES as a differential diagnosis when individuals are diagnosed with seizure disorders, in order to ensure that these individuals are diagnosed correctly at an earlier stage and start treatment as soon as possible. There is also a need for adequately designed controlled studies to evaluate the effectiveness of available methods of treatment of PNES in the South African context.
